# Associated factors and educational and economic inequalities with raised blood pressure in Cambodia: analysis of the data from a national household survey

**DOI:** 10.1186/s12889-026-26522-1

**Published:** 2026-02-09

**Authors:** Maly Phy, Shafiur Rahman, Mahfuzur Rahman, Ada Moadsiri, Sam Ath Khim, Chhinh Liv, Srean Chhim, Savina Chham, Rei Haruyama

**Affiliations:** 1 Present Address: Preventive Medicine Department, Ministry of Health, No. 80 Samdech Penn Nouth Blvd (289), Sangkat Beoungkak 2, Khan Toul Kork, Phnom Penh, Cambodia; 2 Present Address: Graduate School of Health Innovation, Kanagawa University of Human Services, Yokosuka, Japan; 3 Research Center for Child Mental Development, Hamamatsu University School of Medicine, Hamamatsu, Japan; 4 Division of Prevention, National Cancer Center Institute for Cancer Control, Tokyo, Japan; 5 Graduate School of Public Health, St. Luke’s International University, Tokyo, Japan; 6 Present Address: World Health Organization Cambodia, Phnom Penh, Cambodia; 7 Division of Cardiovascular Diseases, National Calmette Hospital, Phnom Penh, Cambodia; 8 School of Public Health, National Institute of Public Health, Phnom Penh, Cambodia; 9 Julius Center for Health Sciences and Primary Care, University Medical Center Utrecht, Utrecht, The Netherlands; 10 Center for Health Research and Policy Support, National Institute of Public Health, Phnom Penh, Cambodia; 11 Centre for Population, Family and Health, University of Antwerp, Antwerp, Belgium; 12 Bureau of Global Health Cooperation, Japan Institute for Health Security, Tokyo, Japan; 13 Noncommunicable Disease Control Project, Japan International Cooperation Agency, Phnom Penh, Cambodia

**Keywords:** Raised blood pressure, Health inequalities, Associated factors, Cambodia

## Abstract

**Background:**

The prevalence of raised blood pressure (RBP) in Cambodia has nearly doubled over the past decade. This study aimed to examine the associated factors and quantify the magnitude of educational and economic inequalities in relation to the prevalence of RBP among Cambodian adults.

**Methods:**

Data were obtained from the 2023 STEPwise approach to noncommunicable disease risk factor surveillance. The study included 3,186 adults aged 18–69 years. Multilevel logistic regression models were used to identify potential associated factors for RBP. The magnitude of educational and economic inequalities was assessed using the regression-based slope index of inequality (SII) and relative index of inequality (RII).

**Results:**

Overall, the prevalence of RBP was 16.2% (95% confidence interval [CI]: 14.5%–18.1%). The main associated factors for RBP were age 40–49 years (odds ratio [OR]: 4.97, 95% CI: 2.51–9.85), 50–59 years (OR:10.67, 95%CI: 5.52–20.62), and 60–69 years (OR:12.92, 95%CI: 6.55–25.48), overweight (OR:1.66, 95%CI:1.19–2.33), obesity (OR: 3.52, 95% CI: 2.38–5.21), and comorbid diabetes (OR: 2.53, 95% CI:1.81–3.54). Female sex (OR: 0.39, 95% CI: 0.25–0.63), current usage of smoking tobacco products (OR: 0.47, 95% CI: 0.27–0.83), adequate consumption of fruits and vegetables (OR: 0.63, 95%CI: 0.46–0.85), and underweight (OR:0.33, 95%CI:0.18–0.61) were associated with reduced risk of RBP. Substantial educational inequality was observed in relation to the prevalence of RBP, with RBP disproportionately affecting individuals without formal schooling at the national (SII: -18.9, 95% CI: -24.80 to -12.90, *p* < 0.001), rural-urban, and regional levels. Nationally, individuals with higher education levels were 67% less likely to have RBP than those without formal schooling (RII: 0.33, 95% CI: 0.17–0.66). Significant absolute economic inequalities in RBP prevalence, to the disadvantage of poor households, were also observed among urban residents (SII: -10.8, 95% CI: -20.10 to -1.50, *p* < 0.05) as well as those living in the plateau and mountain regions (SII: -13.8, 95% CI: -26.10 to -1.40, *p* < 0.05).

**Conclusion:**

RBP remains a major public health challenge in Cambodia, with substantial educational and context-specific economic inequalities. Addressing these social determinants through equity-oriented, context-sensitive interventions is essential to reduce the burden of RBP and prevent cardiovascular diseases in the Cambodian population.

**Supplementary Information:**

The online version contains supplementary material available at 10.1186/s12889-026-26522-1.

## Background

Hypertension (HTN) is characterized by a persistently elevated blood pressure [1]. Approximately 85% to 95% of HTN cases are classified as “primary” or “essential” with no identifiable secondary causes such as renal artery stenosis or pheochromocytoma [2]. Uncontrolled or inadequately controlled, HTN is a major risk factor for cardiovascular diseases, including coronary heart disease, congestive heart failure, ischemic and hemorrhagic stroke, renal failure, and peripheral arterial disease [3].

Worldwide, approximately 1.3 billion people suffered from HTN and 10.5 million died of the condition in 2019 [4, 5]. The prevalence of raised blood pressure (RBP) has doubled in the last 30 years from 650 million in 1990, primarily because of population growth, aging, and lifestyle changes [4, 6, 7]. Notably, more than three-quarters of adults with RBP live in low- and middle-income countries (LMICs), where the majority of deaths from heart disease and stroke also occur [4, 8].

In Cambodia, HTN affects approximately 1.6 million adults aged 30–79 years [6]. The *WHO STEPwise approach to noncommunicable disease risk factor surveillance* (STEPS surveys) reported that the prevalence of RBP among those aged 25–64 years increased from 11.2% in 2010 and 14.5% in 2016 to 19.9% in 2023 [9]. This finding indicates a sharp increase in the incidence of RBP within this age group over the past 13 years. To curb this rising trend, strengthening interventions aimed at preventing modifiable risk factors and promoting the early detection of HTN is essential. Although previous studies in Cambodia have examined the barriers to and determinants of access to HTN screening and treatment services, research on the associated factors for RBP remains limited [10–15]. In 2013, a study identified male sex, older age, daily alcohol consumption, elevated body mass index (BMI), and dyslipidemia as associated factors [16]. However, that study did not assess household- and community-level factors such as income and rural-urban residence. The inclusion of these factors can provide a more accurate understanding of disease risk by moving beyond individual-level characteristics.

Furthermore, studies from both high-income countries and LMICs have reported that inequalities in the prevalence of RBP are influenced by socioeconomic factors such as education and economic status [17–24]. Education may influence health literacy, risk awareness, and health behavior, thereby contributing to differences in the prevalence of RBP, whereas economic disadvantages limit access to nutritional food, health environments, and healthcare, all of which increase the risk of RBP [25, 26]. These socioeconomic factors not only contribute to the risk of developing RBP, but also affect the overall burden of cardiovascular diseases [27, 28]. However, the extent to which socioeconomic factors drive inequalities in the prevalence of RBP in Cambodia remains unclear. Therefore, this study aimed to identify the associated factors and examine the magnitude of educational and economic inequalities in relation to the prevalence of RBP among adults in Cambodia.

## Methods

### Data source

This study used the most recent 2023 data of the STEPS survey in Cambodia, which examined the prevalence of major behavioral and metabolic risk factors and conditions for noncommunicable diseases (NCDs) among the adult population aged 18–69 years. The Ministry of Health led the survey in collaboration with the National Institute of Public Health (NIPH) with technical and financial support from the World Health Organization (WHO). The details of the survey have been described elsewhere [9]. In brief, this cross-sectional survey was conducted across all 25 provinces in the countries in June and July 2023. A multi-stage cluster sampling design was applied to select a nationally representative sample of adults aged 18–69 years. A total of 289 villages, each consisting of 100–200 households, were randomly selected as primary sampling units (PSUs) from 22,949 village-enumeration areas. Then, 15 households per PSU were selected using simple random sampling from a list of households provided by the village chief. From each household, one adult member aged 18–69 years was randomly chosen to participate in the survey.

Because the sampling framework and sample size were determined on the basis of the survey design, no additional sample size calculations were performed. The STEPS survey was designed to be nationally representative, with a sufficient sample size to support disaggregated analyses by sex, age, education, residence, and economic status, thereby ensuring adequate power for the inequality analyses conducted in this study. For statistical power, although the number of households was relatively large, the 212 PSUs, which included 3186 adults aged 18–69 years nested within 3,186 households, provided sufficient statistical information to justify a random intercept model. However, the sample size may limit the ability to detect very small contextual effects.

### Study participants

Among the 4,279 household participants interviewed, we excluded 1,093 participants aged < 18 years or > 69 years and those with missing information for explanatory variables from the analysis (eFigure 1). Overall, the proportion of missing data was low across key variables, except for salt-intake (*n* = 737). A total of 3,186 participants aged 18–69 years were included in the analysis. The characteristics of the excluded samples (due to missing information on covariates) were similar to those of the included participants (eTable 1).

### Outcome variable

The outcome of this study was the prevalence of RBP. RBP was defined systolic blood pressure measurements of ≥ 140 mmHg, or diastolic blood pressure measurements of ≥ 90 mmHg, or current treatment with antihypertensive medication [9, 29]. Each individual underwent three blood pressure measurements using an automated digital monitor, in accordance with the WHO STEPS protocol. The mean of the second and third measurements was used as the final blood pressure value. If only two valid readings were available, the mean value was used. Participants with fewer than two valid readings were excluded from the analysis.

### Explanatory variables

Individual-level, and household-level factors as well as contextual factors previously reported as predictors of RBP were included as explanatory variables [6, 30–33]. Individual-level variables included age (18–29, 30–39, 40–49, 50–59, 60–69 years), since blood pressure increases with vascular aging [34], and sex (male, female), since men generally have higher blood pressure owing to hormonal and behavioral effects [35]. Education level (no formal schooling, primary school completed [up to grade 6], secondary school completed [up to grade 9], high school [up to grade 12], or higher education completed), marital status (currently married, others), and employment status (government employees, non-government employees, self-employed, unpaid) influence health literacy, lifestyle, and access to health care to prevent RBP [36]. Tobacco smoking (never, former, current) and smokeless tobacco use (never, former, current) were included because nicotine causes vasoconstriction and long-term arterial stiffness, leading to increase blood pressure [37]. Alcohol consumption (none, occasionally, daily) elevates blood pressure through sympathetic stimulation and vascular remodeling [38]. Fruit and vegetable intake (low [< 5 servings on average per day], adequate [≥ 5 servings on average per day]) is considered to influence blood pressure through higher potassium, fiber, and antioxidant intake [39]. High salt intake increases blood pressure by causing fluid retention and vascular changes. Reducing salt intake to < 5 g/day has been proven to be cost-effective intervention to prevent HTN and cardiovascular diseases [40]. In the STEPS survey, the estimated daily salt intake was calculated using sodium and creatinine levels in spot urine samples [9]. Because 99.2% of the participants exceeded the WHO’s recommended daily salt intake of 5 g, this variable was categorized into three groups: low (< 5 g/day), high (above 5 g/day but below the average intake of 9.5ga/day [5–9.5 g/day]), and very high (above the average intake [≥ 9.5 g/day]). Physical activity can improve vascular function and reduce body weight, thereby lowering blood pressure, and was categorized as low (< 150 min of moderate-intensity activity per week or equivalent), or adequate (≥ 150 min of moderate-intensity activity per week or equivalent) [41]. BMI (underweight [< 18.5 kg/m^2^], normal weight [18.5 to < 23.0 kg/m^2^], overweight [23.0 to < 27.5. kg/m^2^], obese [≥ 27.5 kg/m^2^]) and comorbid diabetes status (healthy, prediabetes, or diabetes) were included, because obesity and diabetes can contribute to artificial stiffness and endothelial dysfunction, increasing the risk of HTN [42]. For BMI categorization, the cutoff points for Asian populations (≥ 23 kg/m²) were used, given that this threshold has been associated with an increased risk of cardiovascular disease risk factors in a previous Cambodian study [43, 44]. Comorbid diabetes status was categorized as prediabetes when the fasting blood glucose (FBG) level was between 110 mg/dL and 125 mg/dL and as diabetes when the FBG level was ≥ 126 mg/dL or a participant reported taking any medication or insulin for diabetes in the past 2 weeks [9]. The participants were instructed to fast overnight for 8–12 h before blood samples were collected. Fasting status was self-reported, and blood samples were not collected from participants who reported a non-fasting status.

Household economic status was included as household-level variable, and was estimated on the basis of per-capita monthly household income (calculated by dividing the total household income by the number of adult members in the household), and categorized into quintiles [45, 46]. This approach accounts for differences in household size, which can affect each individual’s income, and treats the income variable as ordinal, where a ranked proxy measure of household wealth stratified into quintiles [46, 47]. Community-level variables encompassed the place of residence (rural, urban) and region of residence (central plain, Tonle Sap, coastal and sea, and plateau and mountains) based on the 2019 census [48].

For the analysis of inequality in RBP prevalence, this study focused on the participant’s highest level of education and their household economic status.

### Statistical analysis

The characteristics of study participants were described using percentage distributions, and the prevalence of RBP was calculated as rate per 100 individuals with the 95% confidence intervals (CI). Prevalence of RBP was reported at the national level, and further disaggregated using key covariates (e.g., age, sex, BMI, alcohol consumption, and diet). The chi-squared test was used to assess whether the outcomes differed significantly across the categories of the covariates. In addition, prevalence was calculated in relation to education level and household economic status at the national, regional, and rural-urban levels to visualize socioeconomic and regional inequalities. Sampling weights were considered for all analyses.

Multi-level logistic regression models with a random intercept at the PSU-level were used to identify associated factors for RBP [49, 50]. Odds ratios (ORs) and with 95% CIs were reported. Model 1 was an unadjusted model that provided crude ORs. Model 2 was adjusted for individual-level factors (age group, sex, highest educational level, marital status, employment status, smoking tobacco use, smokeless tobacco use, alcohol consumption, fruit and vegetable consumption, salt intake, physical activity level, BMI, and diabetes status). Model 3 was further adjusted for household-level (i.e., household economic status) and contextual factors (place of residence and region) in addition to individual-level factors. By sequentially adjusting for variables across levels, the modeling approach clarified the distinct influences of personal, household, and regional characteristics on the risk of RBP. Sensitivity analysis was conducted by treating missingness as a separate category within each categorical variable. An additional analysis was performed by excluding participants who had been previously diagnosed with HTN to examine the associated factors with an undiagnosed HTN status. To examine the magnitude of educational and economic inequalities in relation to the prevalence of RBP, regression-based measures-the slope index of inequality (SII) and the relative index of inequality (RII)- were applied at both national and sub-national levels. The SII measures absolute inequality that estimates the difference in RBP prevalence between the most advantaged and disadvantaged groups, considering all intermediate subgroups. An SII value of zero indicates no inequality. A negative value indicates that the outcome is more prevalent among the most-disadvantaged groups, whereas a positive value indicates that the outcome is more prevalent among the most advantaged group. For example, an SII of -10 (the values of SII are multiplied by 100 for better interpretation) would indicate that the prevalence of RBP is 10% higher among the most disadvantaged than in the most advantaged group. In Constrast, RII is a relative measure that expresses the ratio of health prevalence between the most-advantaged and disadvantaged groups, considering all intermediate subgroups [51–54]. An RII value of one (RII = 1) indicates no inequality. RII values greater than 1 (RII > 1) suggest that individuals from the most advantaged groups are more likely to have an outcome of interest, in the case of the RBP, than those from the most disadvantaged group, and vice-versa. For example, an RII of 2.0 would indicate that individuals from the most advantaged group are twice as likely to have RBP than those from the most disadvantaged group. The assessment of both absolute and relative measures are essential to provide a more comprehensive picture of inequalities, and these measures provide complementary insights. In particular, the SII quantifies the size of the gap (percentage-point difference), whereas the RII captures proportional differences relative to group sizes. Divergence between the two measures is possible. For example, when the overall prevalence of RBP is low, absolute differences may appear small (SII close to 0), but relative differences (RII) can still be large.

Data management and statistical analyses were performed using Stata SE version 18. Statistically significant was set at P-value of < 0.05.

## Results

### Characteristics of the study participants

Among the 3,186 participants, 41.3% were aged ≥ 40 years, 47.6% were female, and 70.1% were currently married (Table 1). Nearly half (44.5%) of the participants reported having no formal schooling and approximately 60% were self-employed. The majority of the participants had never used smoking (69.4%) or smokeless tobacco products (94.6%) and reported adequate physical activity (93.5%), but nearly all had high (52.5%) or very high salt intake (47.2%), and approximately 42.7% were overweight or obese. Approximately two-thirds (69.8%) of the participants resided in rural areas.

**Table 1:** Prevalence of raised blood pressure among adults aged 18–69 years in Cambodia

Variables	*N* (weighted %)	Prevalence (95% CI)	*P*-value
Overall	3,186	16.2 (14.5–18.1)	
Individual-level factors			
Age group			< 0.001
18–29 years	385 (38.5)	4.1 (2.1-8.0)	
30–39 years	616 (20.2)	9.6 (6.7–13.5)	
40–49 years	694 (18.7)	22.0 (18.1–26.4)	
50–59 years	808 (15.3)	36.0 (31.8–40.5)	
60–69 years	683 (7.3)	41.9 (37.2–46.8)	
Sex			0.213
Male	1,123 (52.4)	17.2 (14.4–20.4)	
Female	2,063 (47.6)	15.1 (13.4–16.9)	
Highest educational level			< 0.001
No formal schooling	1,868 (44.5)	21.3 (18.7–24.2)	
Primary school completed	599 (23.8)	13.4 (10.0-17.7)	
Secondary school completed	426 (17.7)	11.9 (8.7–16.0)	
High school or higher education completed	293 (14.0)	10.2 (6.7–15.2)	
Marital status			0.052
Currently married	2,187 (70.1)	17.5 (15.6–19.6)	
Others (never married/separated divorced/widowed)	999 (29.9)	13.1 (9.9–17.1)	
Employment status			< 0.01
Government employee	117 (4.0)	20.0 (12.8–29.9)	
Non-government employee	471 (20.3)	10.7 (7.9–14.5)	
Self-employed	1,901 (59.8)	17.0 (14.7–19.6)	
Unpaid (non-paid, students, homemaker, retired, unemployed)	697 (15.9)	19.2 (15.6–23.4)	
Smoking tobacco product use			0.120
Never	2,403 (69.4)	15.8 (13.8–18.0)	
Former user	299 (10.9)	21.5 (16.5–27.5)	
Current user	484 (19.8)	14.7 (11.0-19.4)	
Smokeless tobacco product use			< 0.01
Never	2,855 (94.6)	15.8 (14.0-17.7)	
Former user	89 (1.8)	19.5 (11.1–31.8)	
Current user	242 (3.6)	26.5 (20.5–33.5)	
Alcohol consumption			< 0.01
No	841 (18.0)	19.0 (15.7–22.8)	
Occasionally (< once per day)	2,234 (78.8)	15.1 (13.1–17.2)	
Daily (≥ 1 per days)	111 (3.1)	28.6 (18.8–41.1)	
Fruits and vegetable consumption			< 0.05
Low (< 5 servings on average per day)	2,558 (79.9)	17.0 (15.0-19.2)	
Adequate (≥ 5 servings on average per day)	628 (20.1)	13.0 (10.3–16.4)	
Salt intake			0.304
Low (< 5 g per day)	24 (0.3)	16.8 (5.0–44.0)	
High (5–9.5 g per day)	1,913 (52.5)	15.1 (13.2–17.3)	
Very high (≥ 9.5 g per day)	1,249 (47.2)	17.4 (14.6–20.6)	
Physical activity			0.855
Low (< 150 min of moderate-intensity activity per week or equivalent)	197 (6.5)	15.6 (10.3–23.0)	
Adequate (≥ 150 min of moderate-intensity activity per week or equivalent)	2,989 (93.5)	16.2 (14.5–18.2)	
Body mass index			< 0.001
Underweight (BMI < 18.5 kg/m2)	305 (12.7)	4.0 (2.5–6.4)	
Normal (BMI 18.5-<23 kg/m2)	1,266 (44.6)	12.2 (9.7–15.2)	
Overweight (BMI 23-<27.5 kg/m2)	1,138 (32.8)	20.5 (17.5–24.0)	
Obese (BMI ≥ 27.5 kg/m2)	477 (9.9)	35.7 (29.5–42.5)	
Diabetes status			< 0.001
Healthy	2,594 (89.5)	13.2 (11.5–15.2)	
Prediabetes	202 (4.3)	30.4 (22.6–39.6)	
Diabetes	390 (6.2)	49.2 (42.3–56.2)	

Household-level factors			
Household income			0.863
Poorest quintile	610 (19.4)	17.2 (13.9–21.0)	
Poorer quintile	657 (20.8)	16.3 (12.8–20.6)	
Middle quintile	699 (22.7)	16.1 (12.1–21.2)	
Richer quintile	599 (18.4)	17.1 (13.3–21.6)	
Richest quintile	621 (18.7)	14.3 (11.2–18.1)	

Contextual factors			
Place of residence			< 0.01
Rural areas	1,963 (69.8)	14.7 (12.6–16.9)	
Urban areas	1,223 (30.2)	19.8 (16.7–23.2)	
Region			< 0.05
Central Plain	1,554 (43.4)	19.2 (16.6–22.2)	
Tonle Sap	1,019 (33.7)	14.9 (12.2–18.0)	
Coastal and Sea	190 (5.2)	14.8 (9.4–22.5)	
Plateau and Mountains	423 (17.6)	11.8 (7.9–17.3)	

*CI* Confidence interval

### Prevalence of RBP

The overall prevalence of RBP was 16.2% (95% CI: 14.5–18.1) (Table 1). The prevalence of RBP increased significantly with age and BMI. The prevalence of RBP was relatively higher among participants who were current users of smokeless tobacco product (26.5%, 95% CI: 20.5–33.5), consuming alcohol daily (28.6%, 95% CI: 18.8–41.1), had low fruit and vegetable consumption (17.0, 95% CI: 15.0-19.2), had very high salt-intake (17.4%, 95% CI: 14.6–20.6), and had comorbid prediabetes (30.4%, 95% CI: 22.6–39.6) or diabetes (49.2%, 95% CI: 42.3–56.2).

**Fig. 1:**
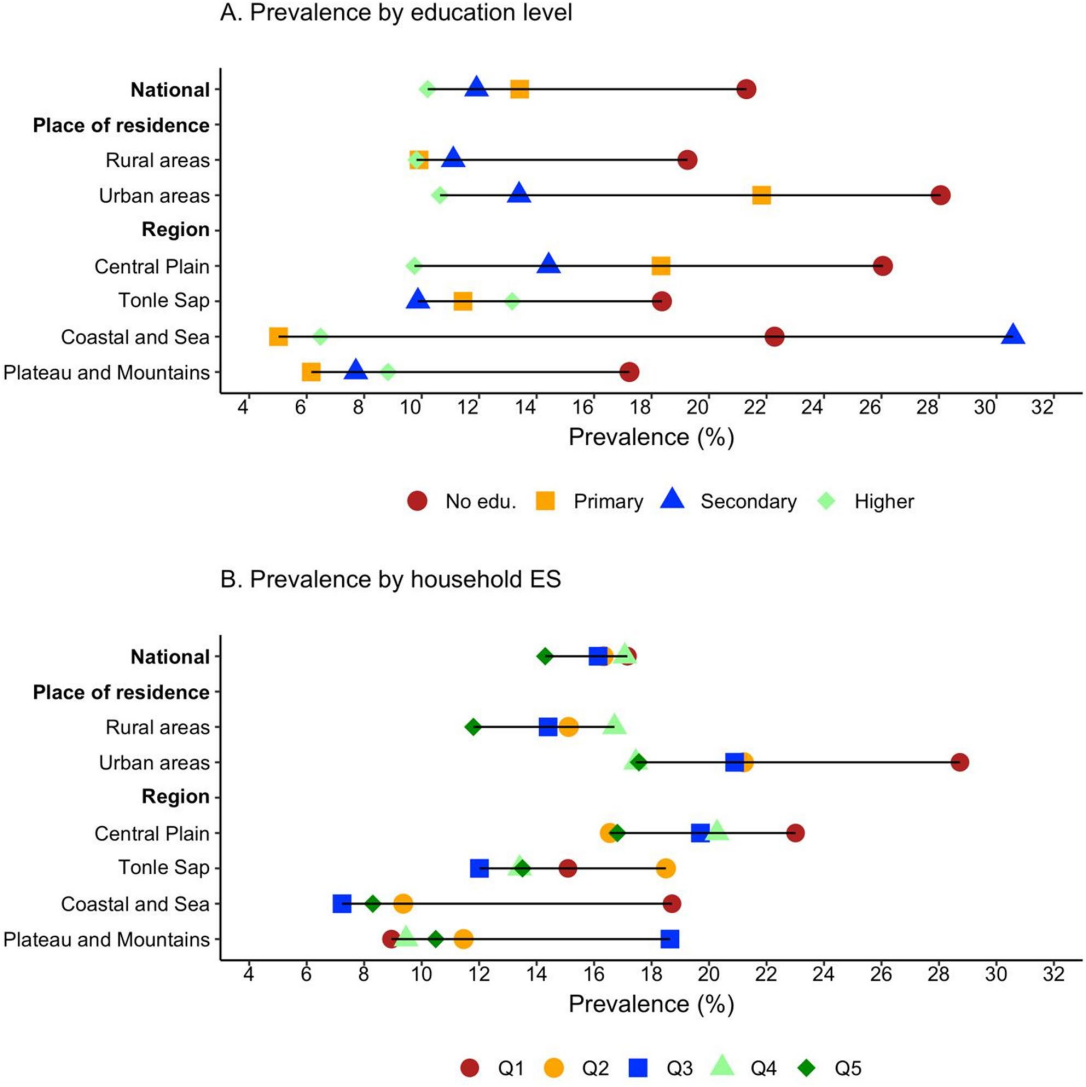
Prevalence of raised blood pressure among adults 18-69 years in Cambodia by education level and household economic status. Panel A: Prevalence by education level. Panel B: Prevalence by household economic status. Notes: No edu., No education; Primary, Primary education; Secondary, Secondary education; Higher, Higher education. Q1, Poorest quintile; Q2, Poorer quintile; Q3, Middle class; Q4, Richer quintile; Q5, Richest quintile

When examining the prevalence of RBP by education level, the prevalence appeared to be the highest among participants with no formal schooling at the national level (21.3%, 95% CI: 18.7–24.2) and in both rural (19.3%, 95% CI: 14.7–24.8) and urban (28.1%, 95% CI: 18.8–39.6) areas (Fig. 1, eTable 2). Similarly, the prevalence of RBP appeared to be the highest among participants from the poorest households, particularly in urban areas (28.7%, 95% CI: 13.2–51.7).

### Associated factors for RBP

Table 2 shows the results of the multi-level logistic regression analysis. After adjusting for individual-, household-, and community-level covariates (Model 3), participants aged ≥ 40 years showed significantly higher odds of RBP than those aged 18–29 years (Table 2). Specifically, the odds of RBP were 4.97 times higher for individuals aged 40–49 years (OR: 4.97, 95% CI: 2.51–9.85), 10.67 times higher for those aged 50–59 years (OR; 10.67, 95% CI: 5.52–20.62), and 12.92 times higher for those aged 60–69 (OR; 12.92, 95% CI: 6.55–25.48). Other factors associated with an increased odds included overweight (OR:1.66, 95%CI: 1.19–2.33), obesity (OR: 3.52, 95% CI: 2.38–5.21) and comorbid diabetes (OR: 2.53, 95% CI: 1.81–3.54). Conversely, female participants (OR: 0.39, 95% CI: 0.25–0.63), current users of smoking tobacco products (OR: 0.47, 95% CI: 0.27–0.83), adequate consumption of fruits and vegetables (OR:0.63, 95%CI: 0.46 − 0.45), and underweight (OR: 0.33, 95% CI:0.18–0.61) showed an inverse association. This inverse association with RBP was not significant for those with high or very high salt-intake (OR:0.78, 95% CI: 0.26–2.31). In our additional analysis, which excluded participants who had been previously diagnosed with RBP, the associations with the use of smoking tobacco products and being underweight were no longer statistically significant (eTable 3). The results remained similar when participants with missing covariate data were included in the model, treating missing data as a separate category within each categorical variable (eTable4).

**Table 2:** Associated factors for raised blood pressure among adults aged 18–69 years in Cambodia

Characteristics	Odds ratio (95% confidence interval)		
	Model 1	Model 2	Model 3
Individual-level factors			
Age group			
18–29 years	1.00	1.00	1.00
30–39 years	2.40 (1.10–5.22)*	2.09 (1.03–4.26)*	2.04 (1.01–4.11)*
40–49 years	6.21 (2.96–13.03)***	5.10 (2.52–10.30)***	4.97 (2.51–9.85)***
50–59 years	12.59 (5.98–26.51)***	11.11 (5.61–22.04)***	10.67 (5.52–20.62)***
60–69 years	16.18 (7.79–33.59)***	13.73 (6.82–27.66)***	12.92 (6.55–25.48)***
Sex			
Male	1.00	1.00	1.00
Female	0.87 (0.67–1.12)	0.39 (0.25–0.62)***	0.39 (0.25–0.63)***
Highest educational level			
No formal schooling	1.00	1.00	1.00
Primary school completed	0.61 (0.40–0.93)*	0.98 (0.59–1.62)	0.93 (0.57–1.52)
Secondary school completed	0.50 (0.32–0.76)**	0.68 (0.40–1.16)	0.64 (0.37–1.10)
High school or higher education completed	0.43 (0.26–0.70)***	0.86 (0.43–1.71)	0.82 (0.41–1.65)
Marital status			
Currently married	1.00	1.00	1.00
Others (never married/separated divorced/widowed)	0.72 (0.51–1.01)	1.54 (1.04–2.30)*	1.54 (1.03–2.31)*
Employment status			
Government employee	1.00	1.00	1.00
Non-government employee	0.47 (0.25–0.87)*	0.61 (0.28–1.37)	0.55 (0.25–1.23)
Self-employed	0.79 (0.47–1.32)	0.85 (0.43–1.68)	0.80 (0.40–1.60)
Unpaid	0.91 (0.55–1.51)	1.10 (0.52–2.32)	1.00 (0.47–2.14)
Smoking tobacco product use			
Never	1.00	1.00	1.00
Former user	1.44 (0.98–2.14)	0.64 (0.39–1.04)	0.65 (0.40–1.06)
Current user	0.86 (0.57–1.31)	0.48 (0.27–0.83)**	0.47 (0.27–0.83)**
Smokeless tobacco product use			
Never	1.00	1.00	1.00
Former user	1.34 (0.79–2.27)	0.75 (0.40–1.39)	0.76 (0.42–1.39)
Current user	1.99 (1.37–2.89)***	0.76 (0.50–1.16)	0.77 (0.50–1.18)
Alcohol consumption			
No	1.00	1.00	1.00
Occasionally (< once per day)	0.76 (0.57–1.03)	1.03 (0.72–1.49)	1.03 (0.71–1.49)
Daily (≥ 1 per days)	1.78 (0.97–3.26)	1.94 (0.98–3.86)	2.03 (1.01–4.11)*
Fruits and vegetables consumption			
Low	1.00	1.00	1.00
Adequate	0.72 (0.54–0.96)*	0.61 (0.45–0.83)**	0.63 (0.46–0.85)**
Salt intake			
Low (< 5 g per day)	1.00	1.00	1.00
High (5–9.5 g per day)	0.90 (0.28–2.88)	0.80 (0.27–2.36)	0.83 (0.29–2.34)
Very high (≥ 9.5 g per day)	1.07 (0.34–3.39)	0.76 (0.25–2.35)	0.78 (0.26–2.31)
Physical activity			
Low	1.00	1.00	1.00
Adequate	1.08 (0.71–1.63)	1.11 (0.68–1.82)	1.10 (0.68–1.77)
BMI			
Underweight (BMI < 18.5 kg/m2)	0.29 (0.17–0.50)***	0.34 (0.19–0.62)***	0.33 (0.18–0.61)***
Normal (BMI 18.5-<23 kg/m2)	1.00	1.00	1.00
Overweight (BMI 23-<27.5 kg/m2)	1.82 (1.31–2.54)***	1.66 (1.17–2.37)**	1.66 (1.19–2.33)**
Obese (BMI ≥ 27.5 kg/m2)	3.97 (2.69–5.85)***	3.56 (2.34–5.40)***	3.52 (2.38–5.21)***
Diabetes status			
Healthy	1.00	1.00	1.00
Prediabetes	2.89 (1.95–4.29)***	1.37 (0.90–2.10)	1.36 (0.89–2.09)
Diabetes	6.25 (4.61–8.47)***	2.52 (1.80–3.54)***	2.53 (1.81–3.54)***

Household-level factors			
Household economic status			
Poorest quintile	1.00		1.00
Poorer quintile	0.97 (0.67–1.40)		1.08 (0.71–1.64)
Middle quintile	0.98 (0.63–1.55)		1.29 (0.78–2.14)
Richer quintile	1.03 (0.69–1.55)		1.23 (0.77–1.97)
Richest quintile	0.83 (0.54–1.27)		0.86 (0.54–1.37)

Contextual factors			
Place of residence			
Rural areas	1.00		1.00
Urban areas	1.44 (1.08–1.92)*		1.14 (0.81–1.62)
Region			
Central Plain	1.00		1.00
Tonle Sap	0.72 (0.55–0.95)*		0.83 (0.59–1.18)
Coastal and Sea	0.71 (0.41–1.23)		0.69 (0.38–1.23)
Plateau and Mountains	0.56 (0.33–0.95)*		0.84 (0.43–1.64)

*BMI* Body mass index, *CI* Confidence interval

*P*-value: *p < 0.05, ^**^p < 0.01, ^***^p<0.001

Model 1: results from separate unadjusted multilevel logistic regression models for each covariate. Each odds ratio represents the crude association between the respective characteristic and hypertension, without adjustment for other variables. Model 2: Adjusted for individual-level factors (participant’s age, sex, highest educational status, marital status, employment status, smoking tobacco product use, smokeless tobacco product use, alcohol consumption, fruits and vegetable consumption, salt intake, physical activity, body mass index, and comorbid diabetes), and Model 3: Further adjusted for household- and contextual-factors (household income quintile, place of residence, and region)

### Educational inequality in relation to the prevalence of RBP

**Fig. 2:**
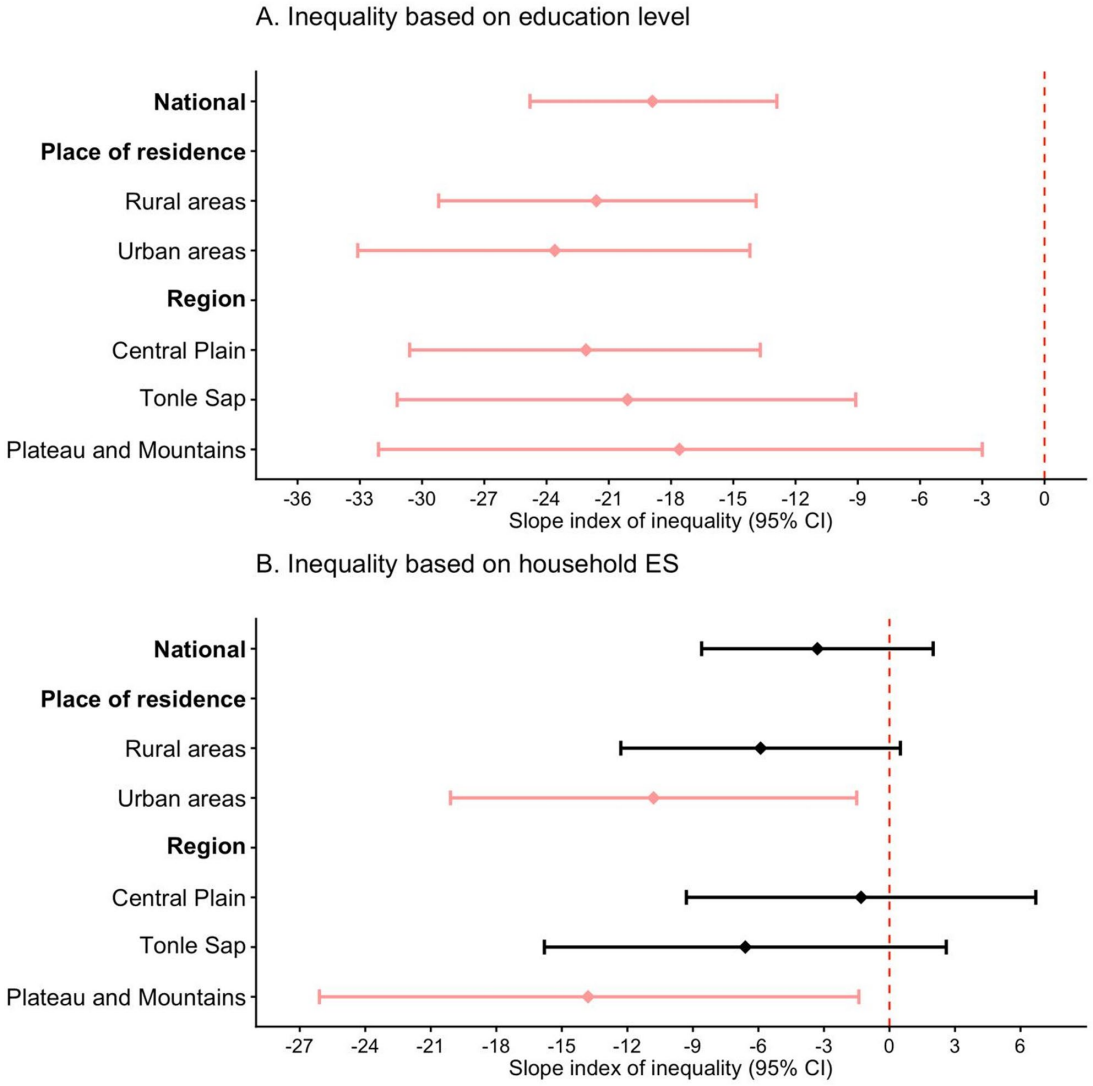
Absolute inequality in relation to the prevalence of raised blood pressure among adults aged 18–69 years in Cambodia. Panel A: Inequality based on education level. Panel B: Inequality based on household economic status. Note: CI, Confidence interval; Inequality analysis was not performed for the coastal and sea region due to small number of samples with outcomes in each subgroup. However, individuals from this region were included in the inequality analyses at the national level and in urban and rural areas

**Fig. 3:**
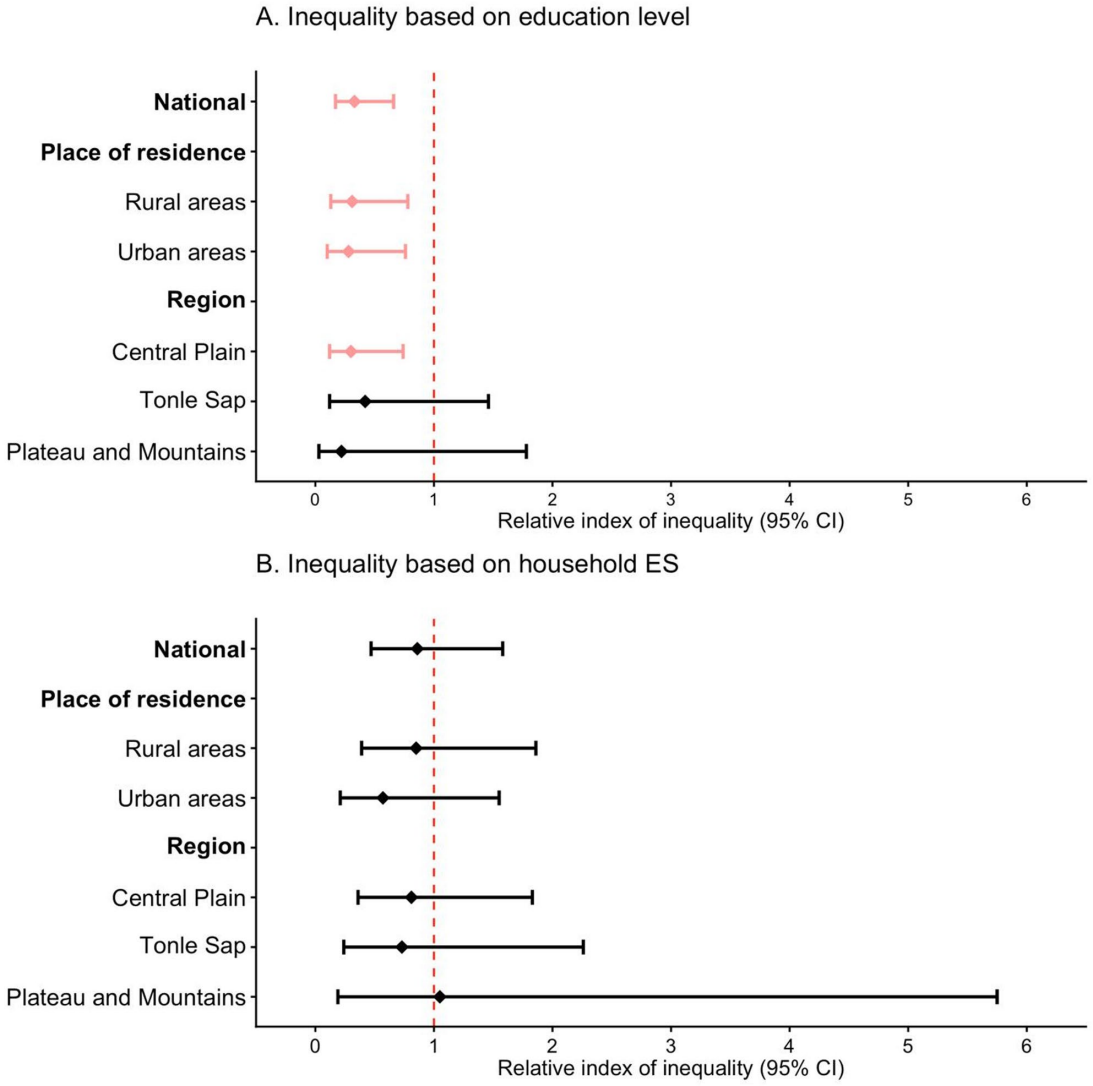
Relative inequality in relation to the prevalence of raised blood pressure among adults aged 18–69 years in Cambodia. Panel A: Inequality based on education level. Panel B: Inequality based on household economic status. Note: CI, Confidence interval; Inequality analysis was not performed for the coastal and sea region due to small number of samples with outcomes in each subgroup. However, individuals from this region were included in the inequality analyses at the national level and in urban and rural areas.

Figure 2 and eTable 5 present the results of the SII describing the magnitude of absolute inequality in relation to the prevalence of RBP, and Fig. 3 and eTable 6 show the results of the RII indicating relative inequality in the prevalence of RBP. At the national level, a significant absolute education-based inequality in the prevalence of RBP was observed, with the prevalence being 18.9% points higher among adults with no education than those with higher education (SII: -18.9, 95% CI: -24.80 to -12.90, *p*<0.001). Such inequality was also significant across rural-urban areas and all geographic regions, except for the coastal and sea regions where the analysis could not be performed because of an insufficient sample size. The relative inequality analysis showed that, at the national level, the highly educated population was 67% less likely to have RBP than individuals with no education (RII: 0.33, 95% CI: 0.17–0.66). This inequality was also observed to a significant extent among rural, urban, and central plain populations.

### Economic inequality in relation to the prevalence of RBP

Absolute economic inequality in relation to the prevalence of RBP between the poorest and richest households was not statistically significant at the national level, although significant economic inequality was observed in urban areas (SII: -10.8, 95% CI: -20.10 to -1.50, *p* < 0.05) and in the plateau and mountain regions (SII: -13.8, 95% CI = -26.10 to -1.40, *p* < 0.05) (Fig. 2 and eTable 5). Relative economic inequality in the prevalence of RBP showed no significance at the national, rural-urban or regional levels (Fig. 3 and eTable 6).

## Discussion

Using nationally representative survey data, this study examined the associated factors of RBP and the extent of educational and economic inequalities in the prevalence of RBP among adults in Cambodia. The overall prevalence of RBP among adults aged 18–69 years was 16.2%. The key factors associated with RBP were age over 40 years, male sex, and obesity (BMI ≥ 27.5 kg/m^2^). Substantial educational inequality in the prevalence of RBP was identified at the national, rural-urban, and regional levels, with RBP disproportionately affecting individuals with no formal schooling. Significant absolute economic inequality, to the disadvantage of poorer households, was also observed in urban areas.

Our findings, which demonstrated significant associations of older age, male sex, and obesity with RBP, are consistent with those of previous studies from Cambodia and other Asian countries [16, 55, 56]. In Cambodia, these associations may indicate that the risk of RBP increases with age due to physiological changes such as arterial stiffening, reduced vascular elasticity, and cumulative exposure to behavioral risk factors [57]. The growing elderly population (age ≥ 60 years) reflects a demographic transition that further heightens the national burden of NCDs, including RBP. Men showed a higher risk of RBP, which may be attributed to cultural norms and sex-based lifestyle differences, including higher rates of tobacco smoking, alcohol consumption, and lower health -seeking behaviors among men [58]. The rising prevalence of overweight and obesity, particularly in urban areas, is linked to dietary transitions, such as increased consumption of processed and high-salt foods, and reduced physical activity associated with urbanization and economic development [59]. Notably, these factors remained robust even after incorporating additional variables into our analysis, such as household economic status and community-level factors, which were not examined in prior studies in Cambodia [16].

Counterintuitively, current usage of smoking tobacco products showed an inverse association with RBP. This may be because individuals with a prior diagnosis of HTN quit smoking after receiving medical advice. Indeed, this inverse association was absent in the additional analysis that excluded individuals with a prior HTN diagnosis. Residual confounding factors, such as the intensity or duration of smoking, may have also influenced the results. Moreover, social desirability bias in self-reported smoking behavior, particularly among women and older adults, could have contributed to underreporting and thus, yielded an apparent protective effect [60]. A similar inverse association was observed between adequate consumption of fruits and vegetables and the prevalence of RBP, consistent with existing evidence that diets rich in plant-based foods and potassium help lower blood pressure [39, 61]. Similarly, the inverse association between underweight and RBP may be explained by lower body fat composition, reduced sympathetic nervous activity, and decreased vascular resistance. Comparable findings have been reported in other Asian populations, where a lower BMI was associated with reduced blood pressure [62]. Conversely, no significant inverse association was observed among those with high or very high salt-intake. This aligns with findings from other low-and middle -income settings, where self-reported salt consumption often fails to accurately reflect true sodium intake because of recall bias and difficulty in estimating salt content in local diets [63–65].

This study observed a marked difference in RBP prevalence across educational levels, with the highest rates observed among adults with no formal schooling and the lowest among those with higher educational attainment. In this study, educational and economic inequalities refer to systematic differences in the prevalence of RBP across ordered categories of education and household income, quantified using SII and RII, and should be interpretated as gradients driven by underlying social determinants rather than as structural inequity measure alone. The SII and RII analyses confirmed significant educational inequalities at the national, rural-urban, and regional levels. Such educational inequality has also been reported in other studies and underscores the critical role of education in shaping health outcomes [18–22]. The observed educational inequalities in RBP prevalence may be related to the participants’ ability to understand and act on health information to prevent lifestyle medications, unhealthy dietary patterns, and reduce access to screening and treatment services. Notably, participants with no formal schooling constituted 44.5% of the participants in our study, more than the 35.1% reported in the 2019 census, likely due to the higher percentage of participants over 50 years old who had limited access to formal education during the years of war and internal conflict [66]. In addition, this discrepancy may be due to (i) differences in population coverage between the STEPS survey (18–69 years) and the census (entire population), (ii) a higher rate of missing data among participants with lower educational attainment, and (iii) possible contextual differences in self-reporting of education between the survey and census settings [67]. Nevertheless, given the higher risk of RBP in older people and Cambodia’s rising life expectancy, educational disparities in the prevalence of RBP are likely to persist or even widen. This situation demands targeted interventions to address the unique barriers faced by individuals with limited or no formal schooling. Health communication strategies should be adapted for low-literacy populations. As a mid- to long-term policy, strengthening health literacy and access to information on preventive health behaviors among school-aged children is essential [68]. The Cambodian Ministry of Education, Youth, and Sport has already incorporated health education related to preventing NCD risk factors, including HTN, into the school curriculum and health education textbooks [69]; however, capacity building of school principals and teachers should be accelerated to maximize this effort.

In our study, economic inequality in relation to the prevalence of RBP was more evident in urban areas and the plateau and mountains regions, where poorer households were disproportionately affected. This pattern aligns with the findings from Kenya and Iran [23, 24]. However, the context in Cambodia differs from that in countries such as India and Nepal, where RBP has been reported to be more prevalent among wealthier groups [19]. These divergent patterns across LMICs may reflect differences in the pace of urbanization, dietary transitions, and access to healthcare. For instance, in India and Nepal, wealthier populations may be more exposed to sedentary lifestyles and high-calories diets, whereas in Cambodia, poorer households may face higher stress, less access to preventive services, and limited opportunities to adopt healthy behaviors. In 2023, Cambodia Ministry of Health launched the Primary Healthcare Booster Framework (PHC-BIF), which aims to strengthen the interaction between communities and health facilities, empowering individuals to better understand and modify their health behaviors to reduce lifestyle-related risks [70]. A systematic review of 14 studies in LMICs found that community health worker-led home- or community-based interventions, such as interpersonal communication on lifestyle modification, blood pressure screening, and support for self-management, significantly improved HTN prevention and control [71]. Overall, the observed educational and economic inequalities in the prevalence of RBP in Cambodia may be related to individuals’ ability to understand and act on health information to prevent lifestyle medications, unhealthy dietary patterns, and limited access to screening and treatment services. These findings have important policy and programmatic implications for future research. For older adults, the interventions should mainly target community-based screening and routine blood pressure monitoring for groups facing a high risk of RBP and its complications, male-focused health promotion campaigns, and strategies to address modified risk factors, such as alcohol consumption and obesity [72]. For individuals with no formal schooling or low educational attainment, health center staffs and village health support groups (VHSGs) could play key roles in delivering this information through interpersonal communication and local networks [73]. At the health system level, the integration of HTN screening and counseling into primary care services, particularly in undeserved rural and disadvantaged areas, could reduce health inequalities [73, 74]. Furthermore, policies to promote healthier food environments (e.g., fiscal measures to reduce the consumption of high-salt or high-sugar products), strengthen health financing mechanisms (e.g., expanding coverage through health equity funds), and increase urban planning initiatives that support physical activity are essential [75, 76].

Although this study is based on a large, nationally representative sample and applied robust analytical methods, including multilevel regression and validated indices of inequality, it has several limitations. First, the cross-sectional design precluded causal inferences between associated factors and RBP. Second, the reliance on self-reported data for key variables, such as dietary intake and alcohol consumption, may have introduced measurement errors owing to recall bias or social desirability bias. Third, some inverse associations (e.g., current tobacco smoking and underweight status with RBP) are likely influenced by residual confounding, behavior change after diagnosis, and reporting bias, and therefore should not be interpreted causally. Fourth, the inequality analysis excluded the coastal and sea region because of an insufficient sample size, which may have limited the generalizability of the findings to that region. However, individuals from this region were included in the inequality analyses at the national level, and in urban and rural areas. Despite these limitations, the use of nationally representative data and robust inequality measures provided valuable insights into the burden of and disparities of associated with RBP in Cambodia. Future research should employ longitudinal designs and include additional socioeconomic and behavioral variables to better elucidate the causal pathways linking education, economic status, and RBP.

## Conclusions

This study demonstrates that RBP is a major public health challenge in Cambodia, driven largely by older age, obesity, and comorbid diabetes, while protective associations were observed among women, individuals with adequate fruit and vegetable intake, and those who were underweight. The findings further highlight significant educational disparities and context-specific economic inequalities, particularly among individuals with no formal schooling and households with lower socioeconomic status in specific regions. Targeted and equity-oriented interventions that focus on high-risk groups, and address social determinants are essential to reduce the burden of RBP and prevent cardiovascular diseases in the Cambodian population.

## Supplementary Information

Supplementary Material 1

Supplementary Material 2

## Data Availability

The same de-identified dataset created by the Ministry of Health Cambodia has been sent to the WHO headquarters in September 2024, to be publicly available on the WHO NCD Microdata Repository ( [https://extranet.who.int/ncdsmicrodata/index.php/catalog/STEPS](https://extranet.who.int/ncdsmicrodata/index.php/catalog/STEPS) ). As of July 2025, the dataset has yet to be shown in the repository; however, it can be shared by the Ministry of Health Cambodia on reasonable request.

## Abbreviations

BMI: Body mass index
CI: Confidence interval
FBG: Fasting blood glucose
HTN: Hypertension
LMIC: Low- and middle-income countries
OR: Odds ratios
PSU: Primary sampling units
RII: Relative index of inequality
SII: Slope index of inequality
STEPS survey: WHO STEPwise approach to noncommunicable disease risk factor surveillance
WHO: World Health Organization
