# Supplementary material for: Associated factors and educational and economic inequalities with raised blood pressure in Cambodia: analysis of the data from a national household survey
Source: BMC Public Health. 2026 Feb 9;26:876. doi: 10.1186/s12889-026-26522-1 (PMC12983574; doi:10.1186/s12889-026-26522-1)
Supplement: Supplementary file 1 — Supplementary Material 1 [file 12889_2026_26522_MOESM1_ESM.docx]

**Supplementary files**

| eTable1: Comparison of background characteristics between included and excluded samples. | | |
| --- | --- | --- |
| Variables | Included samples, N(%) | Excluded samples, N(%) |
| Overall | 3,186 | 1,037 |
| ***Individual-level factors*** |  |  |
| Age group |  |  |
| 18-29 years | 385 (12.1) | 175 (16.9%) |
| 30-39 years | 616 (19.3) | 192 (18.5%) |
| 40-49 years | 694 (21.8) | 213 (20.5%) |
| 50-59 years | 808 (25.4) | 218 (21.0%) |
| 60-69 years | 683 (21.4) | 239 (23.0%) |
| Sex |  |  |
| Male | 1,123 (35.2) | 385 (37.1%) |
| Female | 2,063 (64.8) | 652 (62.9%) |
| Highest educational level |  |  |
| No formal schooling | 1,868 (58.6) | 596 (57.5%) |
| Primary school completed | 599 (18.8) | 173 (16.7%) |
| Secondary school completed | 426 (13.4) | 165 (15.9%) |
| High school or higher education completed | 293 (9.2) | 103 (9.9%) |
| Marital status |  |  |
| Currently married | 2,187 (68.6) | 712 (68.7%) |
| Others (never married/separated divorced/widowed) | 999 (31.4) | 325 (31.3%) |
| Employment status |  |  |
| Government employee | 117 (3.7) | 26 (2.5%) |
| Non-government employee | 471 (14.8) | 199 (19.2%) |
| Self-employed | 1,901 (59.7) | 541 (52.2%) |
| Unpaid (non-paid, students, homemaker, retired, unemployed) | 697 (21.9) | 271 (26.1%) |
| Smoking tobacco product use |  |  |
| Never | 2,403 (75.4) | 750 (72.3%) |
| Former user | 299 (9.4) | 100 (9.6%) |
| Current user | 484 (15.2) | 187 (18.0%) |
| Smokeless tobacco product use |  |  |
| Never | 2,855 (89.6) | 923 (89.0%) |
| Former user | 89 (2.8) | 38 (3.7%) |
| Current user | 242 (7.6) | 76 (7.3%) |
| Alcohol consumption |  |  |
| No | 841 (26.4) | 279 (26.9%) |
| Occasionally (< once per day) | 2,234 (70.1) | 713 (68.8%) |
| Daily (≥1 per days) | 111 (3.5) | 45 (4.3%) |
| Fruits and vegetable consumption |  |  |
| Low (<5 servings on average per day) | 2,558 (80.3) | 851 (82.1%) |
| Adequate (≥5 servings on average per day) | 628 (19.7) | 186 (17.9%) |
| Salt intake |  |  |
| Low (<5 grams per day) | 24 (0.8) | 4 (0.4%) |
| High (5-9.5 grams per day) | 1,913 (60.0) | 197 (19.0%) |
| Very high (≥ 9.5 grams per day) | 1,249 (39.2) | 100 (9.6%) |
| Missing |  | 736 (71.0%) |
| Physical activity |  |  |
| Low (<150 min of moderate-intensity activity per week or equivalent) | 197 (6.2) | 55 (5.3%) |
| Adequate (≥150 min of moderate-intensity activity per week or equivalent) | 2,989 (93.8) | 835 (80.5%) |
| Missing |  | 147 (14.2%) |
| Body mass index |  |  |
| Underweight (BMI <18.5 kg/m2) | 305 (9.6) | 104 (10.0%) |
| Normal (BMI 18.5-<23 kg/m2) | 1,266 (39.7) | 412 (39.7%) |
| Overweight (BMI 23-<27.5 kg/m2) | 1,138 (35.7) | 335 (32.3%) |
| Obese (BMI ≥27.5 kg/m2) | 477 (15.0) | 186 (17.9%) |
| Diabetes status |  |  |
| Healthy | 2,594 (81.4) | 580 (55.9%) |
| Prediabetes | 202 (6.3) | 47 (4.5%) |
| Diabetes | 390 (12.2) | 113 (10.9%) |
| Missing |  | 297 (28.6%) |
| ***Household-level factors*** |  |  |
| Household income |  | <0.001 |
| Poorest quintile | 610 (19.1) | 213 (20.5%) |
| Poorer quintile | 657 (20.6) | 172 (16.6%) |
| Middle quintile | 699 (21.9) | 192 (18.5%) |
| Richer quintile | 599 (18.8) | 166 (16.0%) |
| Richest quintile | 621 (19.5) | 146 (14.1%) |
| Missing |  | 148 (14.3%) |
| ***Contextual factors*** |  |  |
| Place of residence |  |  |
| Rural areas | 1,963 (61.6) | 677 (65.3%) |
| Urban areas | 1,223 (38.4) | 360 (34.7%) |
| Region |  |  |
| Central Plain | 1,554 (48.8) | 504 (48.6%) |
| Tonle Sap | 1,019 (32.0) | 341 (32.9%) |
| Coastal and Sea | 190 (6.0) | 59 (5.7%) |
| Plateau and Mountains | 423 (13.3) | 133 (12.8%) |
| CI: confidence interval  Unweighted percentages are presented in this table.  Two Individuals were excluded from the analysis presented in eTable 5 due to missing information on education (1) and marital status (1), and as a result the characteristics for 1,037 excluded respondents were compared here. | | |

| eTable 2: Prevalence of raised blood pressure among adults aged 18-69 years s in Cambodia by participants’ education level and household  economic status | | | | |
| --- | --- | --- | --- | --- |
| 1. Prevalence by education level | | | | |
| Characteristics | Prevalence (95% confidence interval) | | | |
|  | No education | Primary educated | Secondary educated | Higher education |
| National | 21.3 (17.1-26.3) | 13.4 (9.0-19.6) | 11.9 (7.2-19.0) | 10.2 (5.6-18.0) |
| Place of residence |  |  |  |  |
| Rural | 19.3 (14.7-24.8) | 9.9 (5.6-16.9) | 11.1 (5.8-20.2) | 9.8 (4.1-21.5) |
| Urban | 28.1 (18.8-39.6) | 21.8 (12.3-35.7) | 13.4 (6.0-27.2) | 10.6 (4.4-23.5) |
| Region |  |  |  |  |
| Central Plain | 26.1 (18.9-34.8) | 18.3 (11.2-28.5) | 14.4 (7.4-26.3) | 9.8 (4.0-21.9) |
| Tonle Sap | 18.4 (12.3-26.4) | 11.4 (5.0-24.2) | 9.9 (3.8-23.3) | 13.2 (4.6-32.4) |
| Coastal and Sea | 22.3 (8.0-48.6) | 5.0 (0.2-59.0) | 30.6 (1.8-91.5) | 6.5 (0.5-50.4) |
| Plateau and Mountains | 17.2 (9.3-29.7) | 6.2 (1.5-22.3) | 7.7 (1.7-29.2) | - 1. (1.4-39.5) |

| 1. Prevalence by economic status | | | | | |
| --- | --- | --- | --- | --- | --- |
| Characteristics | Prevalence (95% confidence interval) | | | | |
|  | Poorest | Poorer | Middle class | Richer | Richest |
| National | 17.2 (11.7-24.6) | 16.3 (11.1-23.3) | 16.1 (11.2-22.8) | 17.1 (11.5-24.7) | 14.3 (9.2-21.5) |
| Place of residence |  |  |  |  |  |
| Rural | 15.1 (9.6-23.0) | 15.1 (9.6-22.9) | 14.4 (9.1-22.1) | 16.7 (9.5-27.8) | 11.8 (6.2-21.5) |
| Urban | 28.7 (13.2-51.7) | 21.2 (9.9-39.9) | 20.9 (11.1-35.8) | 17.5 (9.8-29.1) | 17.6 (9.6-29.9) |
| Region |  |  |  |  |  |
| Central Plain | 23.0 (13.6-36.3) | 16.6 (8.8-28.9) | 19.7 (11.9-30.9) | 20.3 (12.0-32.2) | 16.8 (9.4-28.2) |
| Tonle Sap | 15.1 (7.6-27.8) | 18.5 (10.7-30.1) | 12.0 (5.6-24.0) | 13.4 (5.0-31.1) | 13.5 (5.7-28.7) |
| Coastal and Sea | 18.7 (1.7-75.1) | 9.4 (1.0-51.5) | 7.2 (0.7-47.8) | 41.3 (11.3-79.5) | 8.3 (0.6-59.3) |
| Plateau and Mountains | 9.0 (2.4-28.0) | 11.5 (2.9-36.4) | 18.6 (7.7-38.5) | 9.5 (3.0-26.3) | 10.5 (2.9-31.5) |

| eTable 3: Associated factors for raised blood pressure among adults aged 18-69 years in Cambodia after excluding participants previously diagnosed with RBP (N=2,516) | | | |
| --- | --- | --- | --- |
| Characteristics | Odds ratio (95% confidence interval) | | |
|  | Model 1 | Model 2 | Model 3 |
| **Individual-level factors** |  |  |  |
| Age group |  |  |  |
| 18-29 years | 1.00 | 1.00 | 1.00 |
| 30-39 years | 3.34 (1.26-8.81)* | 3.23 (1.34-7.80)** | 3.29 (1.47-7.35)** |
| 40-49 years | 6.88 (2.68-17.68)*** | 6.12 (2.42-15.51)*** | 6.26 (2.70-14.52)*** |
| 50-59 years | 7.87 (2.97-20.90)*** | 7.13 (2.72-18.68)*** | 7.19 (3.00-17.23)*** |
| 60-69 years | 8.01 (3.17-20.27)*** | 6.86 (2.49-18.88)*** | 7.03 (2.86-17.28)*** |
| Sex |  |  |  |
| Male | 1.00 | 1.00 | 1.00 |
| Female | 0.54 (0.38-0.77)*** | 0.37 (0.20-0.68)** | 0.36 (0.20-0.67)** |
| Highest educational level |  |  |  |
| No formal schooling | 1.00 | 1.00 | 1.00 |
| Primary school completed | 0.49 (0.28-0.84)** | 0.58 (0.32-1.08) | 0.59 (0.32-1.09) |
| Secondary school completed | 0.53 (0.27-1.03) | 0.62 (0.28-1.34) | 0.62 (0.29-1.32) |
| High school or higher education completed | 0.53 (0.26-1.10) | 0.75 (0.27-2.05) | 0.81 (0.29-2.22) |
| Marital status |  |  |  |
| Currently married | 1.00 | 1.00 | 1.00 |
| Others (never married/separated divorced/widowed) | 0.72 (0.43-1.21) | 1.72 (0.99-2.97) | 1.75 (1.00-3.08)* |
| Employment status |  |  |  |
| Government employee | 1.00 | 1.00 | 1.00 |
| Non-government employee | 0.64 (0.24-1.74) | 0.68 (0.20-2.31) | 0.66 (0.20-2.15) |
| Self-employed | 0.86 (0.36-2.06) | 0.78 (0.26-2.34) | 0.79 (0.27-2.30) |
| Unpaid | 0.62 (0.23-1.66) | 0.98 (0.25-3.83) | 0.97 (0.26-3.67) |
| Smoking tobacco product use |  |  |  |
| Never | 1.00 | 1.00 | 1.00 |
| Former user | 1.50 (0.89-2.52) | 0.67 (0.36-1.25) | 0.68 (0.36-1.27) |
| Current user | 1.36 (0.77-2.39) | 0.73 (0.35-1.52) | 0.70 (0.32-1.51) |
| Smokeless tobacco product use |  |  |  |
| Never | 1.00 | 1.00 | 1.00 |
| Former user | 0.58 (0.19-1.72) | 0.45 (0.16-1.26) | 0.45 (0.16-1.26) |
| Current user | 0.91 (0.48-1.71) | 0.51 (0.26-0.99)* | 0.50 (0.25-1.01) |
| Alcohol consumption |  |  |  |
| Never | 1.00 | 1.00 | 1.00 |
| Former or occasional drinker | 1.09 (0.63-1.89) | 1.10 (0.58-2.09) | 1.11 (0.58-2.15) |
| Current drinker (≥1 per month) | 4.07 (1.77-9.40)*** | 3.36 (1.40-8.05)** | 3.44 (1.39-8.52)** |
| Fruits and vegetable consumption |  |  |  |
| Low | 1.00 | 1.00 | 1.00 |
| High | 0.54 (0.34-0.87)* | 0.49 (0.31-0.79)** | 0.50 (0.31-0.79)** |
| Salt intake |  |  |  |
| Low (<5 grams per day) | 1.00 | 1.00 | 1.00 |
| High (5-9.5 grams per day) | 2.25 (0.52-9.72) | 1.58 (0.22-11.43) | 1.51 (0.20-11.19) |
| Very high (≥ 9.5 grams per day) | 3.66 (0.83-16.08) | 1.79 (0.23-13.65) | 1.70 (0.22-13.25) |
| Physical activity |  |  |  |
| Low | 1.00 | 1.00 | 1.00 |
| Adequate | 1.14 (0.59-2.19) | 0.87 (0.40-1.88) | 0.85 (0.41-1.79) |
| BMI |  |  |  |
| Underweight (BMI <18.5 kg/m2) | 0.36 (0.18-0.72)** | 0.46 (0.21-1.01) | 0.46 (0.20-1.02) |
| Normal (BMI 18.5-<23 kg/m2) | 1.00 | 1.00 | 1.00 |
| Overweight (BMI 23-<27.5 kg/m2) | 1.82 (1.12-2.97)* | 1.77 (1.09-2.88)* | 1.81 (1.14-2.86)* |
| Obese (BMI ≥27.5 kg/m2) | 3.33 (1.93-5.74)*** | 3.37 (1.93-5.87)*** | 3.49 (2.10-5.82)*** |
| Diabetes status |  |  |  |
| Healthy | 1.00 | 1.00 | 1.00 |
| Prediabetes | 2.54 (1.38-4.67)** | 1.37 (0.74-2.52) | 1.37 (0.73-2.58) |
| Diabetes | 3.90 (2.37-6.40)*** | 1.89 (1.12-3.18)* | 1.92 (1.13-3.28)* |
| **Household-level factors** |  |  |  |
| Household economic status |  |  |  |
| Poorest quintile | 1.00 |  | 1.00 |
| Poorer quintile | 0.95 (0.53-1.70) |  | 0.97 (0.52-1.80) |
| Middle quintile | 0.89 (0.40-1.95) |  | 1.04 (0.45-2.40) |
| Richer quintile | 1.03 (0.56-1.90) |  | 1.09 (0.58-2.03) |
| Richest quintile | 0.80 (0.43-1.49) |  | 0.77 (0.41-1.44) |
| **Contextual factors** |  |  |  |
| Place of residence |  |  |  |
| Rural areas | 1.00 |  | 1.00 |
| Urban areas | 1.09 (0.71-1.67) |  | 0.88 (0.55-1.39) |
| Region^#^ |  |  |  |
| Central Plain | 1.00 |  | 1.00 |
| Tonle Sap | 0.78 (0.50-1.20) |  | 0.80 (0.49-1.33) |
| Coastal and Sea | 1.04 (0.50-2.18) |  | 0.97 (0.48-1.98) |
| Plateau and Mountains | 0.78 (0.33-1.85) |  | 1.05 (0.45-2.44) |
| BMI: body mass index; CI: confidence interval; P-value: *p<0.05; ^**^p<0.01; ^***^p<.001; **Model 1:** results from separate unadjusted multilevel logistic regression models for each covariate. Each odds ratio represents the crude association between the respective characteristic and hypertension, without adjustment for other variables. **Model 2:** Adjusted for individual-level factors (participant’s age, sex, highest educational status, marital status, employment status, smoking tobacco product use, smokeless tobacco product use, alcohol consumption, fruits and vegetable consumption, salt intake, physical activity, body mass index, and comorbid diabetes), and **Model 3:** Further adjusted for household- and contextual-factors (household income quintile, place of residence, and region). | | | |

| eTable 4: Associated factors for raised blood pressure among adults aged 18-69 years in Cambodia after including participants with missing covariate information (N=4,223) | | | | |
| --- | --- | --- | --- | --- |
| Characteristics | Odds ratio (95% confidence interval) | | | |
|  | n (%) | Model 1 | Model 2 | Model 3 |
| ***Individual-level factors*** |  |  |  |  |
| Age group |  |  |  |  |
| 18-29 years | 560 (40.3) | 1.00 | 1.00 | 1.00 |
| 30-39 years | 808 (19.7) | 2.71 (1.33-5.53)** | 2.48 (1.29-4.79)** | 2.45 (1.28-4.66)** |
| 40-49 years | 907 (18.1) | 5.90 (2.95-11.81)*** | 5.33 (2.76-10.31)*** | 5.10 (2.65-9.83)*** |
| 50-59 years | 1,026 (14.6) | 10.98 (5.69-21.18)*** | 10.31 (5.56-19.13)*** | 9.82 (5.35-18.04)*** |
| 60-69 years | 922 (7.3) | 15.48 (7.87-30.46)*** | 13.93 (7.18-27.04)*** | 13.01 (6.69-25.30)*** |
| Sex |  |  |  |  |
| Male | 1,508 (51.3) | 1.00 | 1.00 | 1.00 |
| Female | 2,715 (48.7) | 0.83 (0.66-1.03) | 0.40 (0.28-0.56)*** | 0.39 (0.27-0.56)*** |
| Highest educational level |  |  |  |  |
| No formal schooling | 2,464 (44.5) | 1.00 | 1.00 | 1.00 |
| Primary school completed | 772 (22.6) | 0.56 (0.38-0.81)** | 0.86 (0.56-1.32) | 0.81 (0.54-1.24) |
| Secondary school completed | 591 (18.3) | 0.50 (0.34-0.73)*** | 0.66 (0.41-1.05) | 0.62 (0.38-0.99)* |
| High school or  higher education completed | 396 (14.6) | 0.40 (0.26-0.63)*** | 0.73 (0.41-1.32) | 0.68 (0.37-1.25) |
| Marital status |  |  |  |  |
| Currently married | 2,899 (69.4) | 1.00 | 1.00 | 1.00 |
| Others (never married/  separated divorced/widowed) | 1,324 (30.6) | 0.72 (0.52-1.00)* | 1.52 (1.06-2.18)* | 1.52 (1.05-2.20)* |
| Employment status |  |  |  |  |
| Government employee | 143 (3.5) | 1.00 | 1.00 | 1.00 |
| Non-government employee | 670 (21.0) | 0.59 (0.34-1.04) | 0.75 (0.37-1.53) | 0.69 (0.34-1.41) |
| Self-employed | 2,442 (57.4) | 0.80 (0.50-1.27) | 0.82 (0.44-1.51) | 0.79 (0.43-1.47) |
| Unpaid | 968 (18.1) | 0.90 (0.55-1.46) | 1.09 (0.56-2.12) | 1.03 (0.54-1.97) |
| Smoking tobacco product use |  |  |  |  |
| Never | 3,153 (69.6) | 1.00 | 1.00 | 1.00 |
| Former user | 399 (10.5) | 1.41 (1.00-1.98) | 0.64 (0.43-0.95)* | 0.65 (0.43-0.97)* |
| Current user | 671 (19.8) | 0.87 (0.61-1.24) | 0.48 (0.30-0.76)** | 0.48 (0.29-0.77)** |
| Smokeless tobacco product use |  |  |  |  |
| Never | 3,778 (94.7) | 1.00 | 1.00 | 1.00 |
| Former user | 127 (1.8) | 1.25 (0.79-1.99) | 0.68 (0.41-1.12) | 0.69 (0.42-1.14) |
| Current user | 318 (3.6) | 2.15 (1.58-2.91)*** | 0.83 (0.59-1.18) | 0.84 (0.59-1.20) |
| Alcohol consumption |  |  |  |  |
| Never | 1,120 (19.3) | 1.00 | 1.00 | 1.00 |
| Former or occasional drinker | 2,947 (77.4) | 0.85 (0.64-1.12) | 1.02 (0.74-1.42) | 1.02 (0.73-1.44) |
| Current drinker (≥1 per month) | 156 (3.3) | 2.01 (1.15-3.52)* | 2.06 (1.09-3.89)* | 2.17 (1.13-4.18)* |
| Fruits and vegetable consumption |  |  |  |  |
| Low | 3,409 (80.3) | 1.00 | 1.00 | 1.00 |
| High | 814 (19.7) | 0.65 (0.50-0.86)** | 0.58 (0.44-0.78)*** | 0.59 (0.44-0.79)*** |
| Salt intake |  |  |  |  |
| Low (<5 grams per day) | 28 (0.3) | 1.00 | 1.00 | 1.00 |
| High (5-9.5 grams per day) | 2,110 (43.0) | 1.00 (0.34-2.99) | 0.82 (0.30-2.20) | 0.84 (0.32-2.21) |
| Very high (≥ 9.5 grams per day) | 1,349 (37.4) | 1.17 (0.39-3.47) | 0.76 (0.27-2.17) | 0.78 (0.28-2.16) |
| Missing | 736 (19.4) | 1.25 (0.44-3.58) | 0.83 (0.30-2.27) | 0.87 (0.33-2.31) |
| Physical activity |  |  |  |  |
| Low | 252 (6.3) | 1.00 | 1.00 | 1.00 |
| Adequate | 3,824 (90.8) | 1.06 (0.74-1.51) | 1.14 (0.75-1.74) | 1.11 (0.73-1.69) |
| Missing | 147 (2.9) | 1.42 (0.70-2.90) | 1.43 (0.67-3.09) | 1.39 (0.66-2.95) |
| BMI |  |  |  |  |
| Underweight (BMI <18.5 kg/m2) | 409 (12.5) | 0.31 (0.20-0.48)*** | 0.35 (0.22-0.56)*** | 0.34 (0.21-0.55)*** |
| Normal (BMI 18.5-<23 kg/m2) | 1,678 (44.3) | 1.00 | 1.00 | 1.00 |
| Overweight (BMI 23-<27.5 kg/m2) | 1,473 (31.4) | 1.81 (1.34-2.45)*** | 1.62 (1.16-2.26)** | 1.61 (1.16-2.23)** |
| Obese (BMI ≥27.5 kg/m2) | 663 (11.8) | 3.45 (2.42-4.92)*** | 3.73 (2.50-5.56)*** | 3.70 (2.51-5.44)*** |
| Diabetes status |  |  |  |  |
| Healthy | 3,174 (80.8) | 1.00 | 1.00 | 1.00 |
| Prediabetes | 249 (4.0) | 3.15 (2.12-4.70)*** | 1.53 (1.04-2.24)* | 1.52 (1.04-2.22)* |
| Diabetes | 503 (6.2) | 6.25 (4.75-8.23)*** | 2.68 (1.99-3.60)*** | 2.71 (2.00-3.66)*** |
| Missing | 297 (8.9) |  | 0.97 (0.53-1.78) | 0.96 (0.52-1.77) |
| ***Household-level factors*** |  | 1.30 (0.76-2.22) |  |  |
| Household economic status |  |  |  |  |
| Poorest quintile | 823 (19.8) | 1.00 |  | 1.00 |
| Poorer quintile | 829 (20.4) | 0.89 (0.63-1.24) |  | 0.98 (0.66-1.44) |
| Middle quintile | 891 (21.5) | 0.94 (0.65-1.34) |  | 1.26 (0.86-1.84) |
| Richer quintile | 765 (17.7) | 0.91 (0.63-1.31) |  | 1.08 (0.70-1.65) |
| Richest quintile | 767 (16.6) | 0.89 (0.61-1.29) |  | 0.90 (0.61-1.33) |
| Missing | 148 (4.0) | 0.92 (0.42-2.01) |  | 0.93 (0.44-1.94) |
| ***Contextual factors*** |  |  |  |  |
| Place of residence |  |  |  |  |
| Rural areas | 2,640 (70.0) | 1.00 |  | 1.00 |
| Urban areas | 1,583 (30.0) | 1.42 (1.09-1.85)* |  | 1.18 (0.82-1.69) |
| Region^#^ |  |  |  |  |
| Central Plain | 2,058 (42.7) | 1.00 |  | 1.00 |
| Tonle Sap | 1360 (34.4) | 0.70 (0.54-0.90)** |  | 0.80 (0.59-1.11) |
| Coastal and Sea | 249 (5.4) | 0.73 (0.47-1.11) |  | 0.75 (0.44-1.27) |
| Plateau and Mountains | 556 (17.4) | 0.57 (0.37-0.88)* |  | 0.85 (0.49-1.46) |
| BMI: body mass index; CI: confidence interval; P-value: *p<0.05; ^**^p<0.01; ^***^p<.001; **Model 1:** results from separate unadjusted multilevel logistic regression models for each covariate. Each odds ratio represents the crude association between the respective characteristic and hypertension, without adjustment for other variables. **Model 2:** Adjusted for individual-level factors (participant’s age, sex, highest educational status, marital status, employment status, smoking tobacco product use, smokeless tobacco product use, alcohol consumption, fruits and vegetable consumption, salt intake, physical activity, body mass index, and comorbid diabetes), and **Model 3:** Further adjusted for household- and contextual-factors (household income quintile, place of residence, and region). Two Individuals were excluded from the analysis due to missing information on education (1) and marital status (1). | | | | |

| eTable 5: Absolute inequality in the prevalence of raised blood pressure among  adults aged 18-69 years in Cambodia. | | |
| --- | --- | --- |
| Characteristics | Slope index of inequality (95% CI) | |
|  | Education-based inequality | Economic inequality |
| National | -18.9 (-24.8 to -12.9)^***^ | -3.3 (-8.6 to 2.0) |
| Place of residence |  |  |
| Rural | -21.6 (-29.2 to -13.9)^***^ | -5.9 (-12.3 to 0.5) |
| Urban | -23.6 (-33.1 to -14.2)^***^ | -10.8 (-20.1 to -1.5)* |
| Region |  |  |
| Central Plain | -22.1 (-30.6 to -13.7)^***^ | -1.3 (-9.3 to 6.7) |
| Tonle Sap | -20.1 (-31.2 to -9.1)^***^ | -6.6 (-15.8 to 2.6) |
| Plateau and Mountains | -17.6 (-32.1 to -3.0)* | -13.8 (-26.1 to -1.4)* |
| CI: confidence interval, P-value: *p<0.05, **p<0.01, ***p<.001; Note: Inequality analysis was not performed for Coastal and Sea region due to small number of samples with outcomes in each subgroup. However, individuals from this region were included in the inequality analyses at the national level and in urban and rural areas. | | |

| eTable 6: Relative inequality in the prevalence of raised blood pressure among  adults aged 18-69 years in Cambodia. | | |
| --- | --- | --- |
| Characteristics | Relative index of inequality (95% CI) | |
|  | Education-based inequality | Economic inequality |
| National | 0.33 (0.17 to 0.66)^**^ | 0.86 (0.47 to 1.58) |
| Place of residence |  |  |
| Rural | 0.31 (0.13 to 0.78)* | 0.85 (0.39 to 1.86) |
| Urban | 0.28 (0.1 to 0.76)* | 0.57 (0.21 to 1.55) |
| Region |  |  |
| Central Plain | 0.3 (0.12 to 0.74)^**^ | 0.81 (0.36 to 1.83) |
| Tonle Sap | 0.42 (0.12 to 1.46) | 0.73 (0.24 to 2.26) |
| Plateau and Mountains | 0.22 (0.03 to 1.78) | 1.05 (0.19 to 5.75) |
| CI: confidence interval; p-value: *p<0.05, **p<0.01, **p<.001; Note: Inequality analysis was not performed for Coastal and Sea region due to small number of samples with outcomes in each subgroup. However, individuals from this region were included in the inequality analyses at the national level and in urban and rural areas. | | |
