# Supplementary material for: Associated factors and educational and economic inequalities with raised blood pressure in Cambodia: analysis of the data from a national household survey
Source: BMC Public Health. 2026 Feb 9;26:876. doi: 10.1186/s12889-026-26522-1 (PMC12983574; doi:10.1186/s12889-026-26522-1)
Supplement: Supplementary file 2 — Supplementary Material 2 [file 12889_2026_26522_MOESM2_ESM.docx]

**Supplementary files**

A total of 289 clusters (179 from rural areas and 110 from urban areas) were selected based on the commune databases 2021.

(15 households from each cluster)

1,093 respondents were excluded due to missing information in covariates:

- Respondents aged below 18 years or over 69 years (39)

- Education (1)

- Marital status (1)

- Physical activity (148)

- Wealth quintile (142)

- Diabetes status (283)

- Salt intake level (479)

3,186 respondents aged 18-69 years were included in the final analysis

4,279 households were interviewed

(Rural areas-2,669 and urban areas -1,610)

eFigure 1: Flowchart of participant selection
